# Actinic keratosis modelling in mice: A translational study

**DOI:** 10.1371/journal.pone.0179991

**Published:** 2017-06-29

**Authors:** Arnaud Pillon, Bruno Gomes, Isabelle Vandenberghe, Valérie Cartron, Patrick Cèbe, Jean-Christophe Blanchet, Vincent Sibaud, Nicolas Guilbaud, Laurent Audoly, Laurence Lamant, Anna Kruczynski

**Affiliations:** 1 Pierre Fabre Research Institute, Research & Development Center, Toulouse, France; 2 Department of Dermatology, University Cancer Institute of Toulouse, Toulouse, France; 3 Department of Pathology, University Cancer Institute of Toulouse, Toulouse, France; University of Alabama at Birmingham, UNITED STATES

## Abstract

**Background:**

Actinic keratoses (AK) are pre-malignant cutaneous lesions caused by prolonged exposure to ultraviolet radiation. As AKs lesions are generally accepted to be the initial lesions in a disease continuum that progresses to squamous cell carcinoma (SCC), AK lesions have to be treated. They are also the second most common reason for visits to the dermatologist. Several treatments are available but their efficacy still needs to be improved. The UV-B-induced KA lesion mouse model is used in preclinical studies to assess the efficacy of novel molecules, even though it is often more representative of advanced AK or SCC.

**Objectives:**

Here we report on a translational study, comparing the various stages of AK development in humans and in the UV-B irradiated mouse model, as well as the optimization of photograph acquisition of AK lesions on mouse skin.

**Methods:**

Human and mouse skin lesions were analysed by histology and immunohistochemistry. Mouse lesions were also assessed using a digital dermatoscope.

**Results:**

An histological and phenotypic analysis, including p53, Ki67 and CD3 expression detection, performed on human and mouse AK lesions, shows that overall AK modelling in mice is relevant in the clinical situation. Some differences are observed, such as disorganization of keratinocytes of the basal layer and a number of atypical nuclei which are more numerous in human AK, whereas much more pronounced acanthosis is observed in skin lesion in mice. Thanks to this translational study, we are able to select appropriate experimental conditions for establishing either early or advanced stage AK or an SCC model. Furthermore, we optimized photograph acquisition of AK lesions on mouse skin by using a digital dermatoscope which is also used in clinics and allows reproducible photograph acquisition for further reliable assessment of mouse lesions. Use of this camera is illustrated through a pharmacological study assessing the activity of CARAC^®^.

**Conclusion:**

These data demonstrate that this mouse model of UV-B-induced skin lesions is predictive for the identification of novel therapeutic treatments for both early and advanced stages of the disease.

## Introduction

The development of actinic keratosis (AK), also known as solar keratosis, is a key event for the progression of photodamaged skin to cutaneous squamous cell carcinoma (SCC) [[1], [2], [3]]. These occur primarily on sun-exposed areas and are caused by chronic exposure to ultraviolet (UV) light. Ultraviolet radiation comprises electromagnetic energy covering wavelengths between 100–400 nm. It includes UV-C (100–280 nm), which is absorbed by the atmosphere, but when generated by artificial light sources has profound mutagenic and lethal effects. UV-B (280–320 nm), although representing only ~ 5% of the UV spectrum of solar radiation reaching the surface of the earth, is adept at stimulating cutaneous biological effects, including mutagenic and carcinogenic effects [4]. Cumulative exposure to UV light and increasing life expectancy have resulted in an increased incidence of AK in our aging population, predicting the future impact of AK [5]. In European countries, the AK prevalence among fair-skinned people over 60 years is 20%, increasing to 52% for people over 70 years [4]. If left untreated, AK can progress to invasive SCC [1]. AKs have traditionally been categorized as KIN I or AK I if focal atypia of basal keratinocytes involves only the lower third of the epidermis, KIN II or AK II if atypia affects the two lower thirds of the epidermis or KIN III/ AK III if the atypical cells extend to the upper layers [6]. Traditionally, progression from AK to invasive SCC was considered to occur after an almost complete transformation of the epidermis following the classical pathway from AK I to AK II to AK III. However, recently it was demonstrated that AKs with atypical cells present only in the basal layers (AK I) are the most common precursors of invasive SCC of the skin, therefore suggesting that it is not possible to predict which AKs will progress, regardless of the grade [6]. Furthermore, currently, there are no specific markers to predict tumour aggressiveness and risk of recurrence in patients. If detected and treated in the early stages however, AKs are usually manageable. Several treatments are now approved for managing AKs and their choice is guided by efficacy, adverse effects, cosmetic results and patient compliance [7]. However there is still an unmet need for newer, more efficient and better tolerated treatments or less invasive therapeutic agents. Mouse models are helpful for identification and screening of potentially new treatments. Several studies have previously illustrated the use of UV-B induced skin lesion models to characterize the preclinical activity of drugs dedicated to AK management, such as imiquimod, ingenol mebutate or diclofenac [[8], [9], [10]]. However, these data mainly described mouse models representative of SCC, whereas in this paper we provide additional characterization of this type of model to use it for treating SCC as well as early/intermediate-stage AK. Furthermore, skin tumours in mice in this context have only been assessed clinically or with the help of a commercial off-the-shelf digital camera. The need for monitoring the number and aspect of tumours over time calls for the use of more appropriate standardized imaging devices. For years, dermatologists have turned to specifically designed digital dermatoscopic imaging. The technology of digital videodermatoscopy is mainly used to screen patients’ skin cancers [11] and aims at imaging the skin using a video embedded probe in contact with the patient’s skin. These imaging devices provide a well-defined view of small structures thanks to magnification between 20x and 120x depending on the brand and model.

The purpose of this work was, i) to perform a translational study, that compares the various stages of AK development (early to advanced stages) and SCC in human and mouse UV-B-induced skin lesions and, ii) to optimize photograph acquisition of AK lesions on mouse skin by using a new type of colour-calibrated dermatoscopy allowing reliable comparison of colours and overall aspect over time, therefore improving the use of this UV-B-induced AK mouse model for pharmacological evaluation of new active compounds.

## Materials and methods

### Patients

In compliance with the French law, the Cancer BioBank at IUCT (University Cancer Institute of Toulouse; BB-0033-00014) chaired by Pr. Anne Gomez-Brouchet, and the Oncodermatology specimen collection were declared to the Ministry of Higher Education and Research (DC-2008-463) and a transfer agreement was obtained (AC-2013-1955) after approval by the CPP SOOM I ethics committee (Comité de Protection des Personnes Sud-Ouset et Outre-Mer I) chaired by Me. Denis Benayoun. Patients signed an informed consent under protocol: “LB CE-CBC-KA Pierre Fabre”, provided by the Cancer BioBank at IUCT. They had been previously biopsied for AK/SCC. Only patients with enough material after histological diagnosis were eligible. All patient records and information were anonymized and de-identified prior to analysis.

### Animal ethics statement

Animals were handled and cared for in strict accordance with the Guide for the Care and Use of Laboratory Animals (National Research Council, 1996) and French decree 2013/118 based on European Directive 2010/63/UE, under the supervision of authorized investigators. The study was approved by the CEPC-CEA ethics committee (Centre d'Evaluation Préclinique de Campans—Comité d'Ethique Animale). The national approval number (IACUC) for this committee is C2EA-110, (Comité d'Ethique en Expérimentation Animale—110), and is chaired by Dr. Frédéric Longo. The experimental endpoint for tumor size criterion takes into account the fact that UV-B exposure causes several lesions of different shapes (cones and spheres) on the mouse back. The experiment is stopped when one of the lesions exceeds 1000 mm^3^, or a maximum size of 10 mm (*i*.*e*. a volume of 0.26 cm^3^ and 0.52 cm^3^ for a cone and a sphere, respectively). At each experimental endpoint, mice were ethically euthanized by carbon dioxide inhalation.

### Chronic UV-B exposure in mice

**Fig 1:**
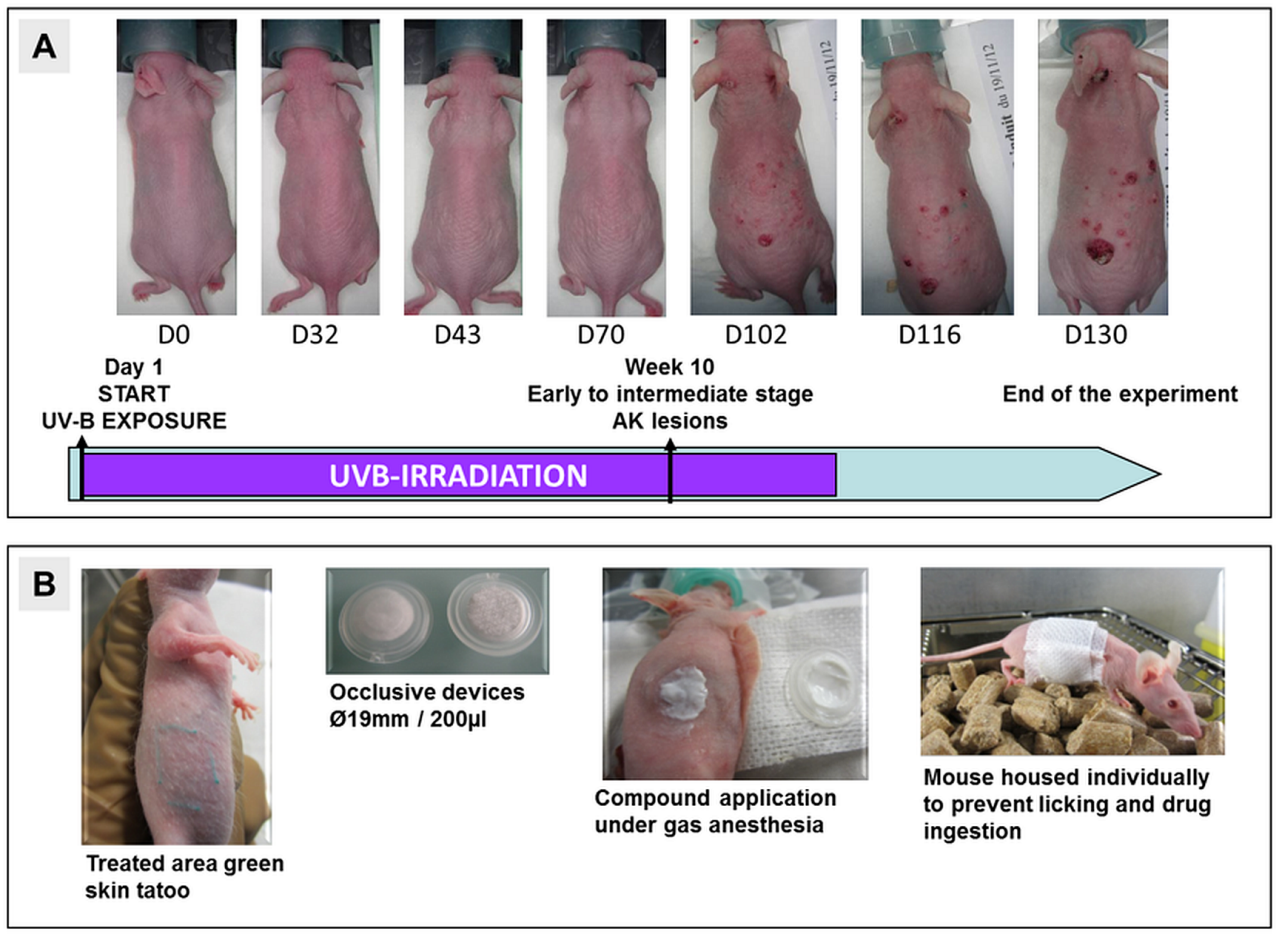
Skin appearance after UV-B irradiation and procedure for topical treatment of skin lesions. (A) Photographs of UV-B-irradiated mouse skin at indicated days post irradiation initiation. (B) Example of tattooed area and of the use of the chamber system to perform topical application.

Hairless SKH-1 mice (Charles River Laboratories, Saint-Germain-sur-L’Arbresle, France) were used for all *in vivo* experiments and were fed with standard chow. Due to the proviral insertion of the murine leukemia virus at the Hr locus which leads to a recessive hypomorphic mutation, these mice do not develop fur but contrary to nude mice are still immuno-competent [12]. SKH-1 mice (6–8 weeks old, weighing 18–20 g) in individual housing (one mouse/cage) were UV-B-exposed every day for 14–15 weeks in a dedicated cabinet which was specifically designed for Pierre Fabre Laboratories, with UV-B exposure of 28 cages simultaneously. Medium wave UV-B lamps T-40.M supplied by Vilber Lourmat (Eberhardzell, Germany), ran from 280 to 320 nm with an energy peak at 312 nm. The irradiation time of a single UVB exposure depends on each lamp’s UV-output and this may decrease slightly over time (UV degradation). To manage this, each lamp was internally calibrated before experiment initiation, using a UVB-specific photo radiometer to adjust the irradiation period. When using new lamps (preheated for 72h to stabilize UV-output), 10–12 min of UV-B exposure per day is necessary to reach the MED (minimal erythemal dose) in SKH-1 mice. The MED of this device was defined as 0.06 J/cm^2^/day. To generate AK lesions and to prevent the risk of skin burn, gradual exposure was performed as follows: 10 days at 0.05 J/cm^2^/day, 10 days at 0.055 J/cm^2^/day and then the MED was applied for the 50 to 80 following days. Skin appearance over time after UV-B irradiation is illustrated in Fig 1A.

### Topical application

After the UV-exposure period, mice developing actinic keratoses were randomized into treatment groups, each consisting of mice with observable skin lesions of comparable number and size. Treated areas were defined and tattooed to be easily identified (Fig 1B). Mice were then treated topically using an occlusive method, employing a ø19 mm chamber system (Hill Top Research St Petersburg, FL, USA) fixed upon treated areas with Tegaderm^™^ film, Omnifix elastic and Elastoplast tape (Fig 1B). All treatment and photographing were performed under isoflurane (1.5%) mixed with air/oxygen (80/20) gas anaesthesia.

### Colour calibrated digital dermatoscope

The digital dermatoscope used in this study is the Colour Calibrated Camera: C-CUBE (Pixience, Toulouse, France). The C-CUBE captures Ultra High Definition (UHD) images with a 5816 dpi density over an area measuring 168 square millimeters in which most tumours fit perfectly. It is piloted using C-CUBE software (Pixience, Toulouse, France).

### C-CUBE software

This software (Pixience, Toulouse, France) provides an easy way to acquire images while following the study. It provides a means of quality control and data management and has a default naming convention. A notable feature of the software is that it helps the user capture images the same way every time by displaying the previously acquired images as guidance for the ongoing observation. This feature guarantees even more reproducibility of the images.

### Histology and immunohistochemistry

Three or four μm-thick sections of formalin-fixed and paraffin-embedded skin biopsies were prepared and stained with hematoxylin, eosin and saffron for histopathological analyses. Immunohistochemical analyses were performed using anti-CD3 (SP7, SpringBioscience, Pleasanton, CA, USA), anti-Ki67 (SP6, Zytomed, Berlin, Germany), and anti-p53 (CM5, Leica Biosystems, Nussloch, Germany) antibodies. Tissue sections (3 μm thick) were cut, deparaffinized, placed in 10 mmol/l citrate buffer (pH 6.0) (for Ki67 and CD3 stainings) or placed in EDTA buffer (pH 9.0) (for p53 staining), and boiled at 96°C for 20 min. Endogenous peroxidase activity was blocked by incubating the sections in 0.3% H_2_O_2_ for 15 min at room temperature (RT). After washing three times with 50 mmol/l phosphate buffer (pH 7.6), the sections were preincubated with 3% bovine serum albumin/5% goat serum in PBS for 1h at RT and were then incubated with the primary antibodies; anti- anti-CD3 (1:100), anti- Ki67 (1:100) and anti-p53 (1:750) for 1h at RT. Subsequently, the sections were incubated with peroxidase-labelled polymer-conjugated secondary antibodies against the primary antibodies for 45 min at RT (1:400 Diagomics D13-D18, Blagnac, France) followed by incubation with 3,3’-diaminobenzidine DAB (DAKO France SAS, Les Ulis, France) using the DAKO Liquid DAB Substrate-Chromogen System for 5 min at RT. The sections were then counterstained with hematoxylin and finally dehydrated and coverslipped.

## Results

### Comparative study of the various stages of AK in human and mouse experimental models

Patient material: twelve patients were selected from a cohort of patients belonging to the oncodermatology department at IUCT (Cancer University Institute of Toulouse), who were previously biopsied for AK/SCC. Selection of various stages of AK, SCC as well as normal skin was possible (Table 1). The average age of patients at the time of diagnosis was 83 years (range 71–98 years).

**Table 1:** Patients and skin lesion characteristics.

PATIENT IDENTIFICATION	AGE^a^ (years) / GENDER	DIAGNOSTIC	SKIN LESION LOCATION
P13.21361	79 / M	AK + SCC	Cheek
P14.1725	88 / M	Infiltrating BCC + AK	Temple
P14.1358	98 / F	AK	Neck
P14.1056	78 / M	AK + SCC	Scalp
P14.1363	87 / F	AK	Inner canthus
P14.1454	87 / F	AK	Eyelid
P14.1521	63 / F	AK	Cheek
P14.1532	83 / M	Acantholytic AK	Forehead
P14.1828	92 / M	Hypertrophic AK	Cheek
P14.1831	93 / F	AK	Shin
P14.1934	80 / M	AK	Shin
P14.1995	71 / F	AK	Eyebrow

^a^mean = 83

Mouse material: SKH-1 mice were UV-B-irradiated daily and skin lesions were sampled at different times.

#### Comparative histological study

**Fig 2:**
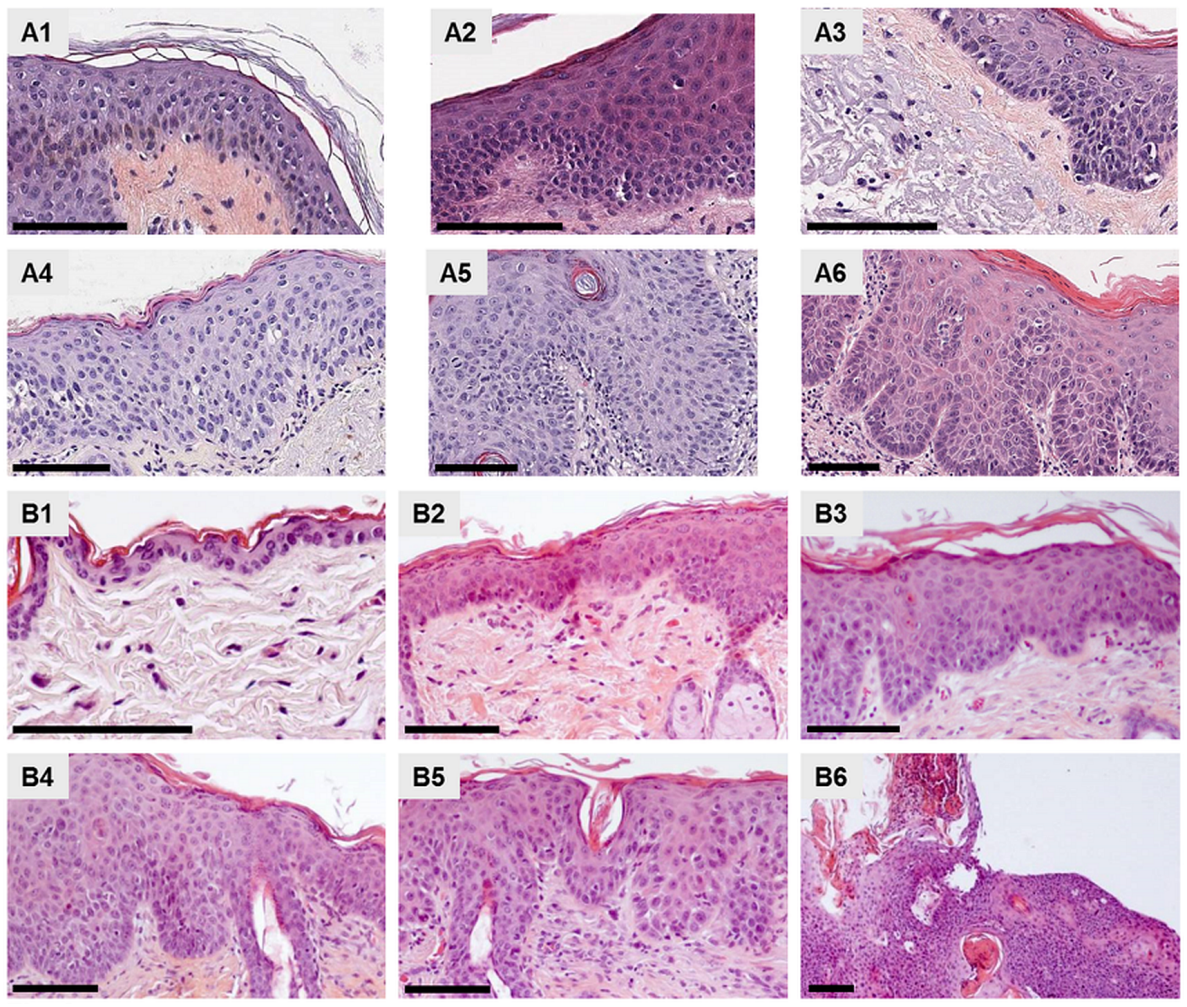
Comparative histology of human and mouse UV-B-induced AK lesions. Hematoxylin and eosin staining. Representative cases: A1) Human normal skin, case 399. A2) Human early-stage AK, case P14-1532. A3) Human early-stage AK, case P14-1358. A4) Human intermediate-stage, case P14-1363. A5) Human advanced-stage AK, case P14-1521. A6) Human advanced-stage AK, case P14-1828. B1) Mouse normal skin, case D181P14. B2) Mouse early-stage AK, case 203P21. B3) Mouse early-stage AK, case D195P16. B4) Mouse intermediate-stage, case 198P39. B5) Mouse advanced-stage AK, case 488. B6) Mouse advanced-stage AK, case D221-1. Scale bars represent 100 μm.

Normal murine skin (Fig 2B1) showed some differences from normal human skin (Fig 2A1). These are mainly a thinner epidermis and a higher number of proliferative cells in the basal layer (Fig 2 and Table 2). Murine skin lesions, sampled on days 67–76 post UV-B irradiation corresponded mainly to early-stage AK-like (Fig 2B2 and 2B3 and Table 2). On day 102, two cases (198P39 / Fig 2B4 and 198P40 / Table 2) were representative of intermediate-stage AK-like with a higher number of atypical keratinocytes in the basal and suprabasal layers, while two other cases (198P43 and 198P44) corresponded to an earlier stage, with fewer less proliferative keratinocytes (Table 2). On day 130 post UV-B irradiation, mouse skin lesions clearly represented advanced-stage AK (Fig 2B5 and 2B6, and Table 2). Overall, the histological comparative analysis showed that AK modelling in mice reflects the clinical situation, with however, some differences (Fig 2A and 2B). In the early to intermediate stages of AK development, abnormal orientation of keratinocytes, no longer perpendicular to the basal layer was frequently observed in human AK but was more rarely seen in the experimental murine AK (Fig 2A and 2B, and Table 2). Unlike the human lesions, murine lesions frequently showed acanthosis already in the early stages of AK development (Fig 2 and Table 2). In both cases, larger nuclei and an increase in the number of mitoses, as compared to normal skin were observed. The advanced stages of AK were characterized in both human and murine cases by a higher number of atypical nuclei and by the presence of mitoses in all layers of the epidermis. Parakeratosis, dyskeratosis or focal points of squamous differentiation and acanthosis were also observed in the advanced forms of murine and human skin lesions (Fig 2A and 2B, and Table 2). An evolution towards cutaneous squamous cell carcinoma was seen in the human P14.1056 sample, as well as in the murine 231–4 case, sampled on day 130 after the start of mice UV-B irradiation (Fig 2A and 2B, and Table 2).

**Table 2:** Comparative histological immunohistochemical analysis of human and mouse UV-B induced AK lesion.

**Patient Id**	**Stage / H&E**	**p53**	**Ki67**	**CD3**
**399**	Normal human skin	-	+	+/-
**V160915**	Normal human skin	-	+	+/-
**P14,1831**	Early-stage AK	+	+/- A few postive cells	+/-
**P14,1725**	Early-stage AK + infiltrating BCC Atrophic epidermis	Heterogenous staining with ++ areas	++	+/-
**P14,1532**	Early-stage AK Acantholytic	+/-	++	+
**P14,1358**	Early to intermediate-stage AK Atrophic skin with limited epidermis Anormal cell alignment in basal layer Large atypical nuclei	+/- Heterogenous and weak staining	+++ Postitive staining in basal and suprabasal layer	Only focal points of inflammatory reaction
**P14,1934**		-	+	+
**P14,1995**		-	Heterogenous staining: + to ++	+/-
**P14,1363**	Intermediate-stage AK Anormal cell alignment in basal layer Large atypical nuclei	Negative	+++	Weak infiltrate
**P14,1056**	Advanced AK with focal points of SCC	Negative: AK areas +: SCC areas	++ Heterogenous staining, with positive cells detected above the basal layer, in normally non-cycling cell areas	T cell infilration ++
**P14,1454**	Advanced AK With some squamous differentiation areas A few mitoses outside of the basal layer	+	++	+
**P14,1521**	Advanced AK With some squamous differentiation areas A few mitoses outside of the basal layer	++	+	+
**P14,1828**	Advanced AK With some squamous diffrentiation areas Amonalies in the supra basal layer	+++	Heterogenous staining: + to +++	++ very inflammatory
**P13,21361**	Advanced AK With some squamous differentiation areas	-	+	+
**Mouse Id**	**Day after the start of irradiation Stage / H&E**	**p53**	**Ki67**	**CD3**
**D181-p14**	Normal mouse skin	-	+/ -	+/-
**D186-p03**	Normal mouse skin	- (rare + cells)	not done	++
**203P21-L1**	D67-early stage AK Acanthosis, thickening of the stratum granulosum Dyskeratotic cells in the mucous body	heterogenous + to +++	+++	+
**203P22-L2**	D67-early Stage AK Abnormal cell orientation in the basal layer Large nuclei, thicker stratum garnulosum, Thicker stratum corneum without nuclei (orthokeratosis)	++ to +++	+	+/- (zone+)
**195P15**	D76-early stage AK Acanthosis, Hypergranulosis, orthokeratotic dyskeratosis, Mitoses in the basal layer, larger nuclei	heterogenous + to +++	++	+ (zones++)
**195P17**	D76-early stage AK Acanthosis	heterogenous + to +++	++	+
**195P16**	D76 Early-stage AK Larger nuclei in the basal layer, abnormal organization of the basal layer, acanthosis, parakeratotic dyskeratosis, hypergranulosis, mitosis increase	heterogenous + to +++	++	+
**198P43**	D102 Less advanced-stage AK, less proliferative, acanthosis, Orthokeratotic dyskeratosis	heterogenous + to +++	++	+
**198P44**	D102 Less advanced-stage AK, less proliferative, acanthosis, Orthokeratotic dyskeratosis	heterogenous + to +++	+ (zones ++)	+ (zones++)
**198P39**	D102 Intermediate-stage AK Acanthosis, parakeratotic dyskeratosis, atypical nuclei in the basal, suprabasal layers as well in the mucous body	++ to +++	++	+/- (zones +)
**198P40**	D102 Intermediate-stage AK Acanthosis, parakeratotic dyskeratosis, atypic cell increase, mitosis increase in basal and suprabasal layers	heterogenous + to +++	+ (rares zones ++)	+
**231–4**	D130 Advanced-stage AK + SCC Acanthosis, atypical keratinocytes and mitoses in the various epidermis layers	+++	++ (zones +++)	+ (zones++)
**D221-1**	D130 Advanced-stage AK Acanthotic epidermis; atypical keratinocytes and mitoses in the various epidermis layers	++	+++	+

Scoring: +/-: rare positive cells, +: less intense staining and about 15% positive cells, ++: intense staining and about 30% positive cells, +++: intense staining and about 50% positive cells

Number of mice in each subgroup: Normal (no AK): 2 mice / Early stage AK: 5 mice / Intermediate-stage AK: 4 mice / Advanced-stage AK: 2 mice

Overall the characteristics of the various stages of AK development in the UV-B-irradiated mouse model are very close to those of human AK, with an increase in the size of nuclei, the number of atypical nuclei and mitoses, a modification of keratinocyte distribution in skin layers. However acanthosis is much more abundant in mice than in humans and appears at early stages.

#### Comparative immunohistological study

**Fig 3:**
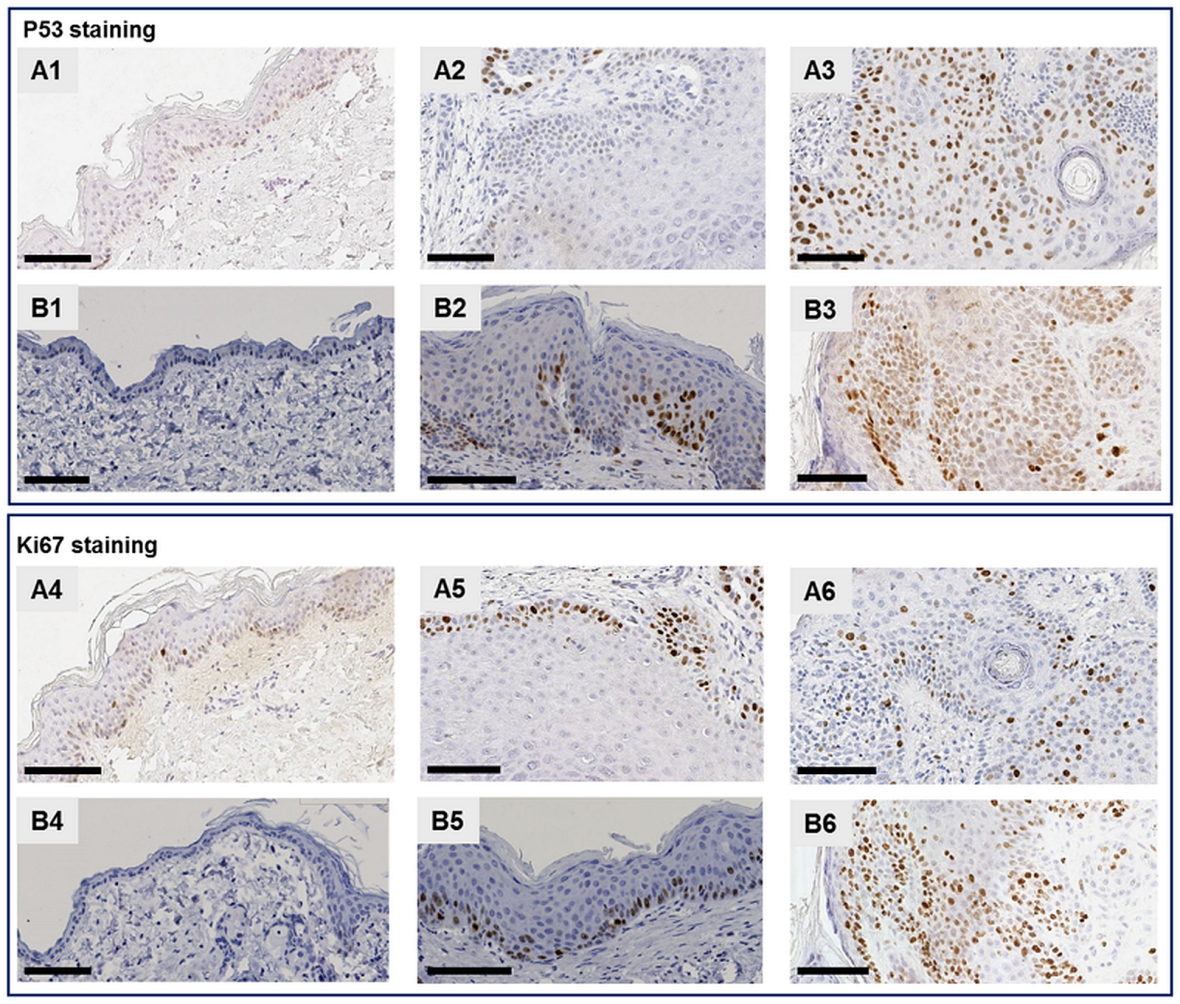
Phenotypic analysis of human (A) and mouse UV-B-induced (B) AK lesions. P53 and Ki67 staining. Histological sections of normal human skin (pictures labelled “A”) and normal murine skin (pictures labelled “B”) as well as human and murine AK lesions (labelled “A” and “B” respectively) at various stages were stained with P53 (pictures with 1, 2 or 3 numbers) or Ki67 antibodies (pictures with 4, 5 or 6 numbers). Representative cases: Human, normal skin, case 399: A1) p53 staining, A4) Ki67 staining. Human, early-stage AK, case P14-1532: A2) p53 staining, A5) Ki67 staining. Human, advanced-stage AK, case P14-1521: A3) p53 staining, A6) Ki67 staining. Mouse, normal skin case D181P14: B1) p53 staining, B4) Ki67 staining. Mouse, early-stage AK, case D195P16: B2) p53 staining, B5) Ki67 staining. Mouse, advanced-stage AK, case D-221-1: B3) p53 staining, B6) Ki67 staining. Scale bars represent 100 μm.

P53 staining was observed on keratinocyte nuclei, in 8 of the 12 human skin lesions and in all murine skin lesions, particularly in the basal layer but also in the other layers of the epidermis, especially in the advanced forms (Fig 3A2, 3A3, 3B2 and 3B3 and Table 2). A heterogeneous pattern of expression, in terms of staining intensity was more frequently observed in the early-to-intermediate human and murine AK cases (Table 2). Furthermore, as AK progressed to advanced stage or to SCC, a higher number of keratinocytes showed positive p53 staining in both human and murine lesions (Table 2 and Fig 3).

Overall, the majority of both human and murine skin lesions showed a higher number of proliferating keratinocytes in the basal layer and in some cases in the suprabasal layer of the epidermis than in normal skin (Fig 3A5, 3A6, 3B5 and 3B6 and Table 2). A Ki67 score of ++ or +++ was recorded in 8 of the 12 human skin lesions and a Ki67 score of ++ or +++ was also recorded in 8 of the 11 murine skin lesions (Table 2).

Because the AK immune infiltrate mainly consists of T cells, the CD3 marker was used to characterize immune infiltration in AK and SCC specimens. CD3-positive T-cells were detected in a higher number of human cases than in murine cases (Table 2 and S1 Fig). In fact, very few murine skin lesions showed a positive CD3 infiltrate (Table 2 and S1 Fig). A CD3 score of + was recorded in 5 of the 12 human specimens, while inflammatory T cell infiltration (++ score) was observed in 2 of the 12 cases (Table 2).

### Pharmacological validation of the mouse model of UV-B induced AK lesions

**Fig 4:**
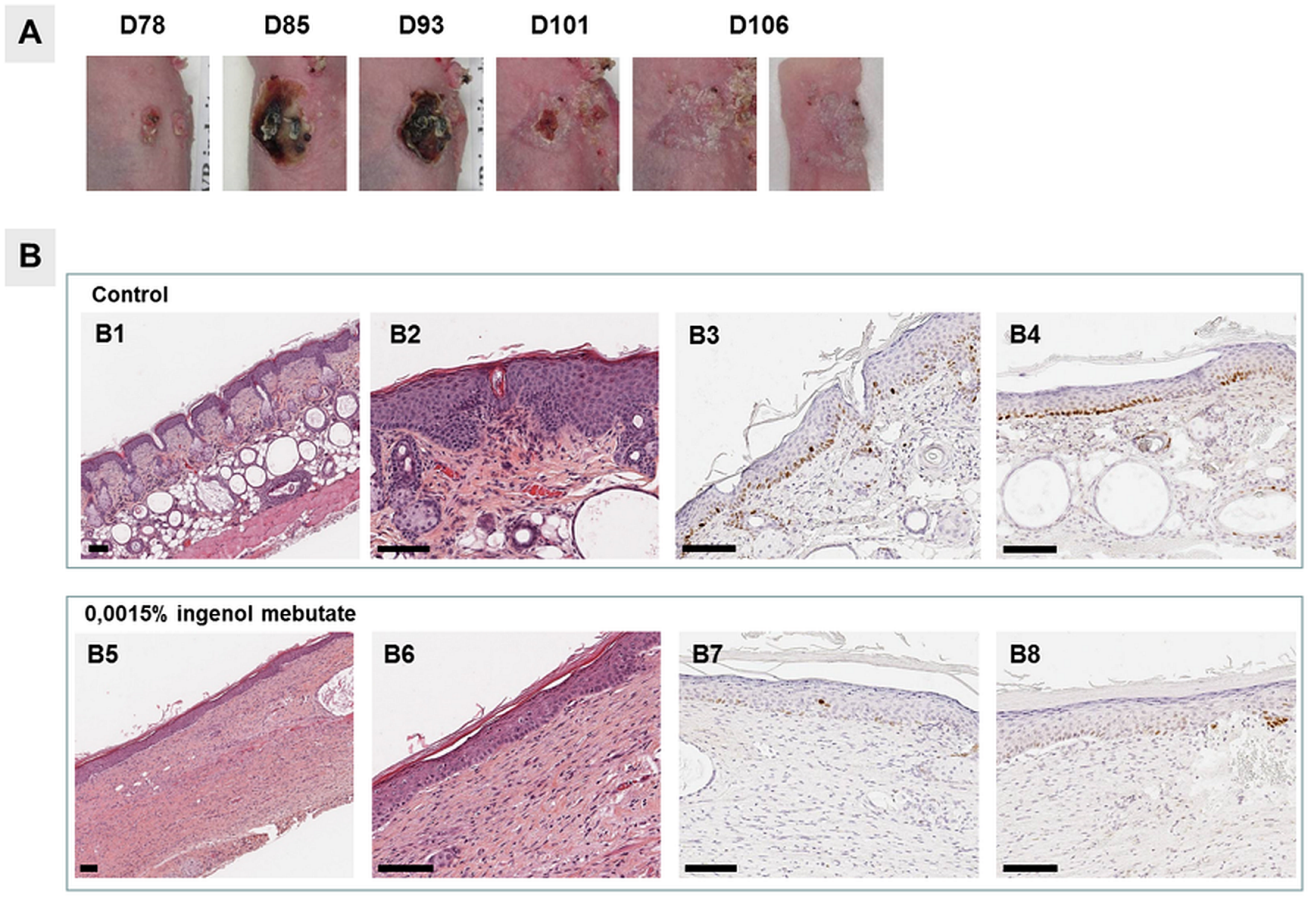
Ingenol mebutate inhibits UV-B-induced AK lesions. Mice were exposed to UV-B irradiation for 74 days, and then topically treated for 5h with either 0.015% ingenol mebutate or control vehicle (5% DMSO, 70% Glycerol and 25% H_2_O) on day 78 after irradiation initiation. A) Skin appearance after ingenol mebutate treatment, at days indicated after irradiation initiation. B) Histology of UV-B-irradiated mouse skin topically treated with either control vehicle or 0.015% ingenol mebutate. B1-B2) Hematoxylin and eosin staining of control vehicle-treated skin. B3) Ki67 staining of control vehicle-treated skin. B4) p53 staining of control vehicle-treated skin. B5 and B6) Hematoxylin and eosin staining of 0.015% ingenol mebutate-treated skin. B7) Ki67 staining of 0.015% ingenol mebutate-treated skin. B8) p53 staining of 0.015% ingenol mebutate-treated skin. Experiments involved three mice per experimental group. Scale bars represent 100 μm.

At the time of our study, ingenol mebutate was one of the most recently approved drugs for the topical treatment of AK. To further confirm the pharmacological validation of this AK model we evaluated the efficacy of 0.015% ingenol mebutate (PICATO^®^) used as a topical occlusive application to treat early-stage murine AK lesions induced by 74 days of UV-B irradiation. Occlusive topical field-directed treatment of AK lesions with 0.015% ingenol mebutate over 5h on day 78 resulted in a 100% cure rate in the three treated mice; defined in Fig 4A as no visible AK lesions on day 106 after the start of UV-B irradiation. At 7 days post ingenol mebutate treatment, clear signs of haemorrhage and necrosis in the skin lesions were evident, and then eschars formed over the lesion sites, which resolved 28 days post treatment. On day 106 skin was fully regenerated (Fig 4A). Histological analysis of the control non-treated AK skin lesions confirmed the expected epidermal thickening that arises after chronic UV-B irradiation (Fig 4B1 and 4B2). On day 106, less proliferative keratinocytes, reflected by a lower number of cells showing Ki67 and p53 staining, were clearly present to reconstitute normal epidermis (Fig 4B7 and 4B8). However a thinner epidermis and an altered dermis, exhibiting modified skin muscle and adipocyte density were noted and no sebaceous gland, or hair follicle could be observed (Fig 4B5 and 4B6). The main adverse effect observed with ingenol mebutate treatment was severe erosion of the treated area.

### Optimization of photograph acquisition of mouse AK lesions using a calibrated digital dermatoscope–Application to a pharmacological study: Effects of CARAC^®^ 0.5% fluorouracil (5-FU) cream on mouse AK

**Fig 5:**
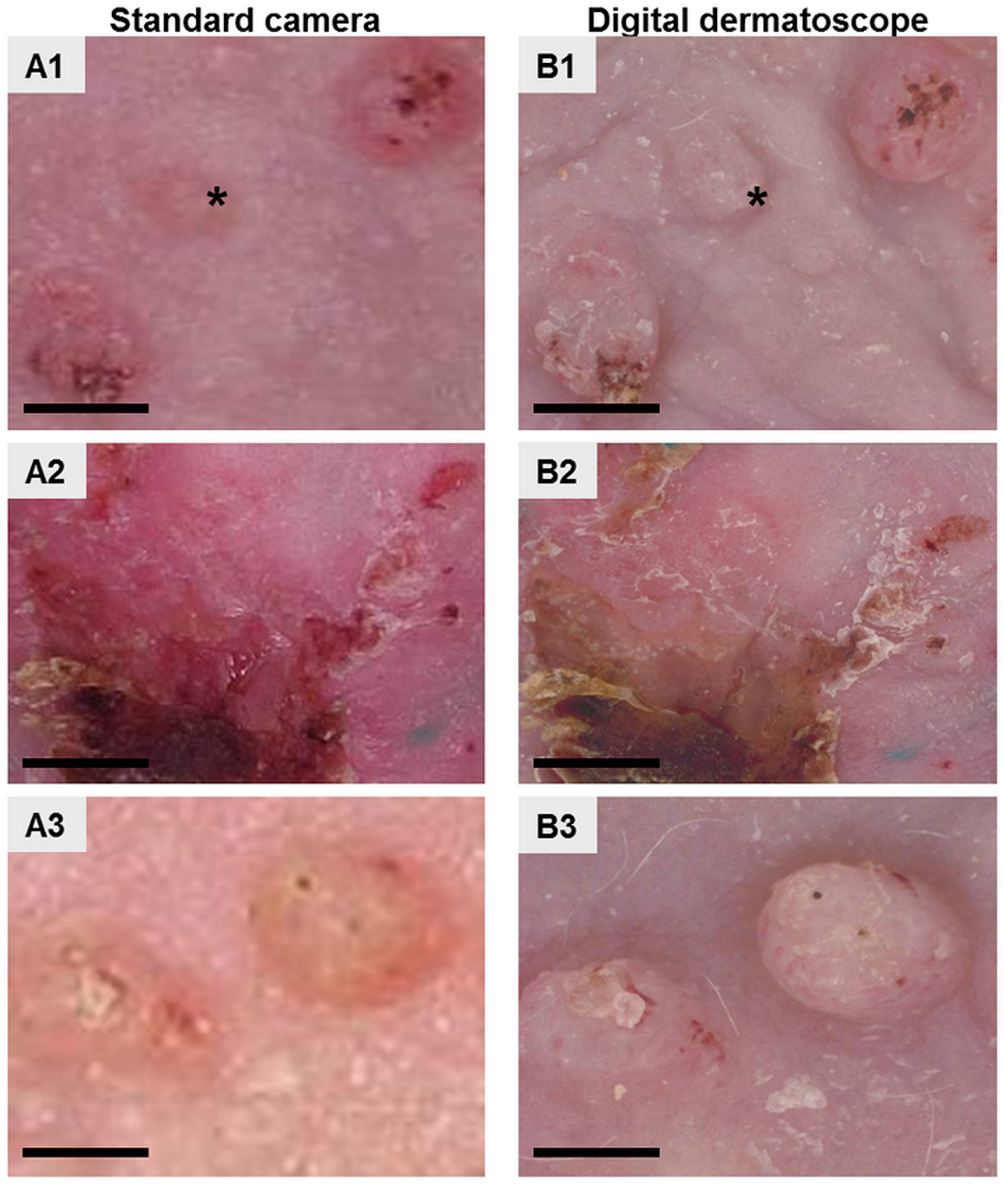
Comparison of macroscopic skin lesions. Photographs taken with either a standard camera (A1-A3) or a calibrated digital dermatoscope (B1-B3). Mice were exposed to UV-B irradiation for 108 days and photographs were taken on the indicated days after irradiation initiation. Scale bars represent 500 μm.

Assessment and preclinical diagnosis of UV-B-induced mouse lesions are mainly based on morphological observation using photographs and histological analysis. Taking pictures of mouse skin lesions with a standard camera is known to result in inter-picture variability in terms of lightness and colour and therefore it is often difficult to reproduce the detection of early-stage AK lesions. Since our objective was to model the various stages of AK development, we decided to optimize photograph acquisition by using a calibrated digital dermatoscope, also used in clinics. We illustrated the use of this calibrated digital dermatoscope through a pharmacological study assessing the effects of 0.5% 5-FU (CARAC^®^), one of the well-known reference topical treatments for AK [13], on mouse UV-B induced skin lesions. Mice were treated topically with 0.5% 5-FU for two cycles of 30h and pictures of skin lesions were taken with either the calibrated digital dermatoscope or a standard camera (Camera Canon Power Shot SX230 HS) at different times after the start of treatment. Overall the pictures of AK taken using the calibrated digital dermatoscope showed better contrast and lightness homogeneity than those taken with the standard camera (Fig 5). Colour and lightness parameters were reproducible for each picture taken with the digital dermatoscope over time, after the start of UV-B irradiation (see Fig 5B1–5B3), whereas differences were visible in the pictures taken with the standard camera (see Fig 5A1–5A3). For example, the picture taken on day 140 with the standard camera was much paler than the one taken on day 112 (see Fig 5A1 and 5A3). The most striking difference between the two cameras was related to colour contrast of the picture. The use of the digital dermatoscope allowed for individual distinguishing of the three-dimensional skin lesions and therefore clearly identification of small early-stage lesions that could have been missed with the standard camera (see Fig 5A1 and 5B1).

**Fig 6:**
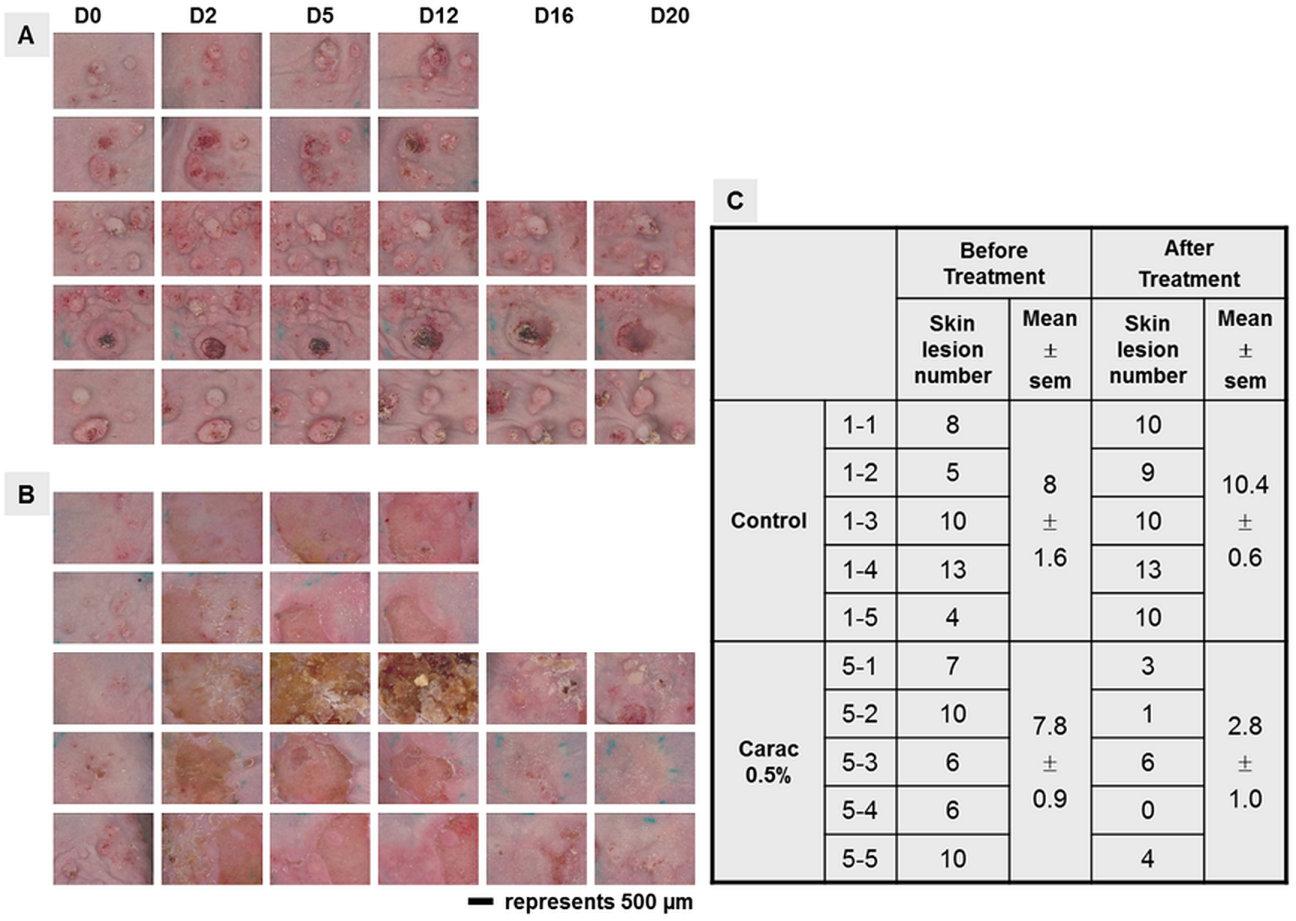
0.5% 5-FU inhibits UV-B-induced AK lesions. Mice were exposed to UV-B irradiation for 74 days, then topically treated for 30h with either 0.05% 5-FU or control vehicle (5% DMSO, 70% Glycerol and 25% H_2_O), starting on day 78 after irradiation initiation. The chamber systems containing the 0.05% 5-FU cream were then removed for 20h, and mice were again treated topically for 30 additional hours. Skin appearance after either control vehicle (A) or 5-FU (B) treatment, at indicated days after treatment initiation. Scale bars represent 500 μm. C) Assessment of the number of skin lesions in control vehicle and 5-FU treated mice, before and after treatment (on day 28 after treatment initiation). Experiments involved five mice per experimental group.

Overall the digital dermatoscope ensured perfectly comparable images over time. We used this digital dermatoscope to investigate the effects of 0.5% 5-FU on UV-B-induced AK lesions (Fig 6A and 6B). 0.5% 5-FU decreased the number of AK lesions by 64%, as assessed by analysis of photographs on day 28, whereas the number of skin lesions in the non-treated mice group increased by 25% (Fig 6C).

## Discussion

AKs are skin lesions which are precursors to SCCs on sun-exposed areas through genomic perturbations due to UV radiation and may develop progressively to invasive SCCs. AK treatment is therefore mandatory. Understanding human neoplasia relies on, but has also been limited by, the experimental models used to study tumour initiation and progression. Therefore, correlation of experimental observations made in murine models and in human tissue is of prime importance for demonstrating relevance to human disease. Mouse models, most notably those employing the SKH-1 hairless immunocompetent mouse, proved to be useful and allowed the identification of critical molecular and biologic changes that trigger skin tumour development [[14], [15]]. They are also commonly used to assess the preclinical efficacy of novel treatments on SCC however, rather than on AK lesions [[10]; [9], [8], [16]].

The goal of the study presented here was to provide a detailed translational study, comparing the various stages of AK development in humans and in mice, as well as optimization of photograph acquisition for a more reliable pharmacological assessment of the activity of novel treatments.

Mice treated with a UV-B protocol are recognized as the most relevant models of human skin diseases since they develop AK-like lesions and SCC’s resembling those seen in humans, although differences exist, such as the thickness of the skin, which is much thinner in mice than in humans [17]. UV-B lamps used in our device cover 280 to 320 nm with an energy peak at 310 nm. There is a possibility of these lamps being contaminated by UV-C wavelengths [18]. Unfortunately, we did not monitor the spectra in the UVC range, and therefore, cannot exclude the UV-C effect. This problem will be addressed in our future research. To completely avoid UV-C emission it could be beneficial to cover UV lamps with Kodacel filters that act as cut-off filters removing all UV wavelengths below 290 nm.

In this study, we characterized and compared various stages of skin lesion development in UV-B irradiated mice and in a series of human cases. Three grading scales were established: early-stage AK, intermediate-stage AK and advanced-stage AK, which often included SCC areas. In fact the coexistence of AK lesions and SCC on the same individual is frequently observed in 72 to 92% of cases in the clinical situation, and it is commonly admitted that 60% of cutaneous carcinoma arise from AKs [5]. Our classification of the various AK stages here is very similar to the three levels of histological characterization, AK I, AK II and AK III based on the grade of atypical keratinocytes [[19], [5]]. Some differences between murine and human cases were mainly observed in the early stages of AK development: keratinocyte disorganization was more frequently observed in human cases and more significant acanthosis was seen in the earliest stages of mouse skin lesion development. Another main difference between human AK and the mouse UV-B-induced AK is that human skin lesions, having been exposed to sun, arise for the most part in elderly individuals, having aged skin, which constitutes structural differences when compared to the skin of the 4–6 week-old mice used in our study. A way of optimizing this model could be to establish AK lesions in older mice and to irradiate them with UV-B over a longer period of time. However overall, AK development in our mouse model is relevant to human cases, with the same characteristics of an increasing number of atypical keratinocytes progressively present in the different layers of the epidermis and thickening of the stratum corneum. Furthermore this translational study enabled us to define the appropriate experimental conditions for establishing either early, intermediate or advanced stage AK, or an in situ SCC model for pharmacological studies, depending on whether the intended therapy aims at preventing SCC development or treating SCC.

Considering the recent evolution of AK grading and SCC risk, which suggests that the risk of transformation of an individual AK into invasive SCC cannot be ascertained on the basis of clinical or histopathological features [6], it would certainly be of value to further investigate this model by homogenizing groups of mice according to AK grade (AK I, II or III), assessing whether they evolve differently and comparing data to the clinical situation.

In order to provide an additional pharmacological validation of this mouse UV-B-induced AK model, the effects of ingenol mebutate were assessed. The application of 0.015% ingenol mebutate to mouse UV-B-damaged skin resulted in a marked reduction of skin lesions, with adverse events generally mild to moderate, corresponding to previously published preclinical and clinical reports [[20], [9]]. In this paper we included another pharmacological study using 0.5% fluorouracil to illustrate the optimization of photograph acquisition; in these two pharmacological studies, the 2 agents, ingenol mebutate and 0.5% fluorouracil were given to mice with early-stage AK and intermediate-stage AK, respectively. We did not treat mice with advanced AK since the initial objective of these studies was to prevent the evolution of AK. Clearly, it is to be expected that application of the same treatment would be more effective on early AK but in this case since the treatments were different, we could not compare them.

Assessment of the number of AK lesions is based on the pictures taken; therefore quality, sensitivity, contrast, homogeneity and reproducibility are critical. Here we showed that the use of the calibrated digital dermatoscope allows reproducible photograph acquisition, with better contrast and homogenous colours, compared to pictures taken with a standard camera. Video dermatoscopy has much higher pixel density compared to standard images obtained with off-the-shelf digital cameras. This device uses a patented lighting principle (WO/2015/075177) [21] to produce homogeneous illumination without any glare which is quite difficult to achieve using standard cameras. Moreover, since the images are taken in contact with the skin, the lighting is perfectly controlled and cannot be affected by any surrounding brightness. This allows for colour calibration of the device from the RGB colour space of the sensor to the RGB colour space and then to the CIELab colour space [[22], [23]]. This calibration process guarantees reliable colour measurements and reproducible images across C-Cube probes. Finally, since this device uses fixed zoom and focus settings, it ensures the best image quality even when handled by untrained personnel and guarantees an identical scaling factor across images. Therefore the use of this imaging device optimizes assessment of the number of skin lesions, including the detection of small lesions or the residual disease after treatment or at relapses. The development of software to automatically quantify skin lesions, on the basis of the pictures taken by the calibrated digital dermatoscope could be considered a way of further improving the pharmacological assessment process.

In conclusion, the overall data presented here provide elements of optimization for the use of the UV-B induced AK mouse model and a more detailed translational comparison of the various stages of AK development in mice and in humans.

## Supporting information

S1 Fig Phenotypic analysis of human and murine UV-B-induced AK lesions. CD3 staining. Representative cases. A1) Human, normal skin, case 399. A2) Human, early-stage AK, case P14-1532. A3) Human, advanced-stage AK, case P14-1521. B1) Mouse, normal skin, case D181P14. B2) Mouse, early-stage AK, case D195P16. B3) Mouse, advanced-stage AK, case 488.
(TIF)
