# Supplementary figures and images for: Actinic keratosis modelling in mice: A translational study
Source: PLoS One. 2017 Jun 29;12(6):e0179991. doi: 10.1371/journal.pone.0179991 (PMC5491107; doi:10.1371/journal.pone.0179991)

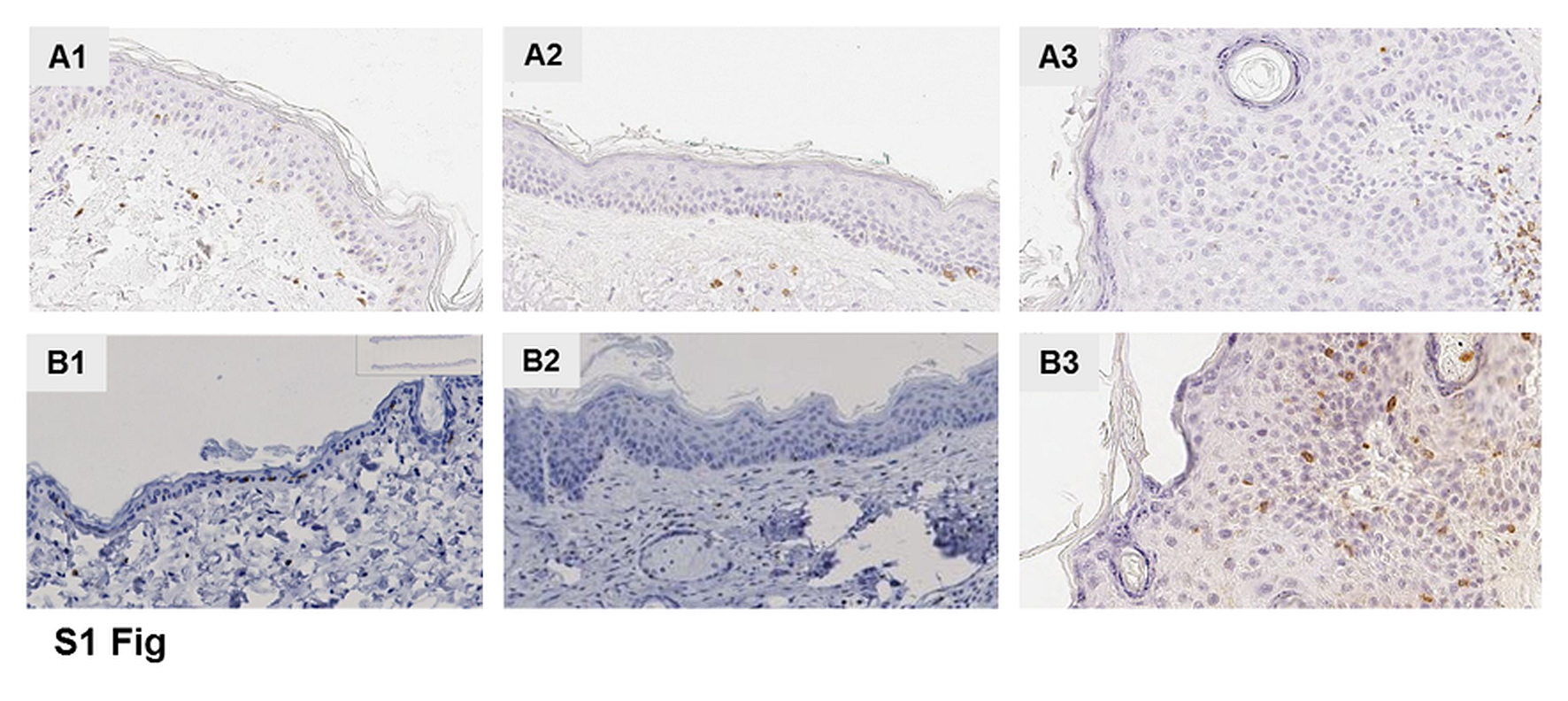

Supplement: S1 Fig — CD3 staining. Representative cases. A1) Human, normal skin, case 399. A2) Human, early-stage AK, case P14-1532. A3) Human, advanced-stage AK, case P14-1521. B1) Mouse, normal skin, case D181P14. B2) Mouse, early-stage AK, case D195P16. B3) Mouse, advanced-stage AK, case 488. (TIF) [file pone.0179991.s001.tif]
